# ABC Transporters, APOE, CYP46A1, and LRP1 Gene Polymorphisms as Markers of Dementia Development in Patients with Hyperlipidemia

**DOI:** 10.3390/ijms262110759

**Published:** 2025-11-05

**Authors:** Marta Machowska, Jerzy Leszek, Maja Rączy-Krzemianowska, Beata Tomasiewicz, Magdalena Hurkacz, Małgorzata Rąpała, Janusz Piechota, Krystyna Głowacka, Anna Wiela-Hojeńska

**Affiliations:** 1 Department of Clinical Pharmacology, Wrocław Medical University, 50-556 Wrocław, Poland; magdalena.hurkacz@umw.edu.pl (M.H.); krystyna.glowacka@umw.edu.pl (K.G.); 2 Department of Psychiatry, Wroclaw Medical University, 50-367 Wroclaw, Poland; jerzy.leszek@umw.edu.pl; 3 Clinical Department of Internal Medicine, 4th Military Clinical Hospital with Outpatient Clinic, SP ZOZ, 50-981 Wrocław, Poland; mraczy@4wsk.pl (M.R.-K.); btomasiewicz@4wsk.pl (B.T.); 4 Department of Pediatric Surgery, T. Marciniak Lower Silesian Specialist Hospital, 54-049 Wrocław, Poland; mrapala@poczta.onet.pl; 5 Amplicon Ltd., 54-413 Wrocław, Poland; jkpiechota@amplicon.pl

**Keywords:** ABCA1, ABCB1, APOE, CYP46A1, LRP, dementia, hyperlipidemia, polymorphism, markers

## Abstract

In an aging society, solving problems associated with the diagnosis and treatment of dementia-related diseases represents a serious challenge. The aim of the study was to evaluate the possibility of applying molecular biology methods to test polymorphisms recognized in the global literature as potentially useful in assessing the risk of developing dementia in a group of patients with hyperlipidemia. A sample of 203 patients: 109 diagnosed with both dementia and hyperlipidemia, 94 with hyperlipidemia, and 101 individuals as an allele frequency control group—were genotyped. Additional data about cognitive decline and neuropsychological assessment were collected. Among all the studied polymorphisms, the frequency of the *ABCA1* rs2230806 polymorphism differed between the analyzed groups. The GG genotype (*p* = 0.0002, RR = 3.22, CI = 1.63 ÷ 6.37) and the G allele (*p* = 0.0007, RR = 1.53, CI = 1.19 ÷ 1.97) were more frequent in patients diagnosed with dementia, specifically in those with Alzheimer’s disease. Furthermore, the GG genotype was more common in individuals with a shorter disease duration and lower scores on the Montreal Cognitive Assessment (MoCA) scale, and consequently, with greater cognitive function deficits during early stages of the diagnostic process. *ABCA1* rs2230806 genotyping is a potential marker for the early identification of dementia risk in patients with hyperlipidemia, which supports the validity of exploring options for incorporating diagnostics based on molecular biology methods.

## 1. Introduction

The World Health Organization (WHO) defines dementia as a set of symptoms of a progressive or chronic nature, in the course of which cognitive functions deteriorate to a greater extent than in the case of the biological aging process. Nearly 10 million new cases are diagnosed every year [1]. In 2021 cardiovascular disease (CVD)/ischemic heart disease and stroke were, respectively, first and third cause of death worldwide [2]. The existing literature indicates that shared risk factors such as body mass index (BMI), hypertension, diabetes, and dyslipidemia can be affected by genetics. Numerous convergent genes are involved in the disease progress of both morbid conditions [3]. The growing importance of molecular diagnostics in clinical practice might be a useful tool to understand pathogenesis and simultaneously a chance to modify patient lifestyle before the prodromal phase. According to a recent report based on analysis of nearly half a million cases, hyperlipidemia diagnosed at a younger age results in higher risk of all-cause dementia (HR 1.46 in diagnosis < 50 years) [4]. Moreover, lifestyle modification after CVD diagnosis reduces the risk of developing dementia [5]. Conclusions from observational studies and genotyping trials provide evidence that genetic markers are needed in public health policy for prevention.

Table 1 presents the genes selected for analysis based on their alignment with lipid metabolism processes and the development of dementia.

To follow this line, we conduct *ABCA1*, *ABCB7*, *ABCB1*, *APOE*, *CYP46A1*, and *LRP1* genotyping to compare allele frequency in three groups of patients diagnosed with (1) dementia with coexisting hyperlipidemia or (2) hyperlipidemia, or who were (3) healthy controls, to clarify their role in dementia development and assess their potential for use as early markers of dementia among patients with dyslipidemia.

## 2. Results

### 2.1. Baseline Characteristics

A total of 304 participants, 109 dementia and hyperlipidemia patients, 94 with hypercholesterolemia, and 101 individuals as an allele frequency control group, were included in the study. No significant difference was observed in sex distribution. Patients with both dementia and hyperlipidemia were older than those with hyperlipidemia (70.8 (±11.5) vs. 64.1 (±12.9). Dementia and hyperlipidemia participants had a lower burden of comorbidities. Both diabetes and hypertension were more frequently observed in the hyperlipidemia group (*p* < 0.05). The mean age of dementia diagnosis was 65.8 (±9.8) and duration was on average 7.8 (±2.4). Mini–Mental State Examination (MMSE) and MoCA scores were, respectively, 18.1 (±4.0) and 19.0 (±4.1). In the hyperlipidemia and dementia group, laboratory workup showed significantly increased cholesterol, high-density lipoprotein (HDL), low-density lipoprotein (LDL), and triglyceride levels (*p* < 0.05 in all parameters). Table 2 presents a comparison of the characteristics between the dementia and hyperlipidemia group and the hyperlipidemia group.

### 2.2. Polymorphisms

First, we assessed differences in genotype frequency in all experiment groups: dementia and hyperlipidemia, no dementia with hyperlipidemia, and controls (Table 3). The distributions of the *ABCA1* (rs2422493), *ABCA7*, *ABCB1*, *APOE*, *CYP46A1*, and *LRP1* genotypes were not significantly different. The *ABCA1* (rs2230806) genotype was not evenly distributed between groups (*p* = 0.004). Further analysis confirmed the existence of differences in the distribution of genotypes between the dementia and hyperlipidemia versus hyperlipidemia groups, with a more frequent prevalence of the GG genotype (*p* = 0.0002, RR = 3.22, CI = 1.63 ÷ 6.37) and G allele (*p* = 0.0007, RR = 1.53, CI = 1.19 ÷ 1.97) in patients with dementia. This genotype was related to an increased risk of Alzheimer’s disease diagnosis (*p* = 0.006). We also compared the occurrence of genotypes in males and females, but no differences were observed.

**Figure 1:**
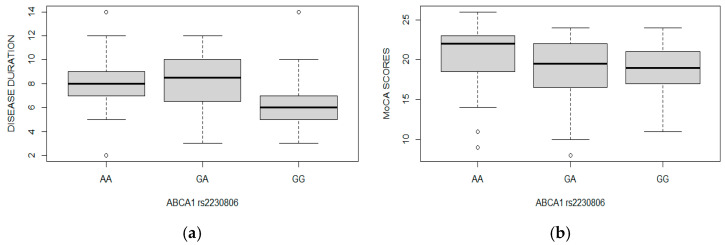
(**a**) Boxplot illustrates the distribution of individual *ABCA1* (rs2230806) genotypes and the duration of the disease; (**b**) boxplot illustrates the relationship between the distribution of *ABCA1* (rs2230806) genotypes and MoCa scores. Whiskers indicate the variability outside the upper and lower quartiles; circles mark outliers.

Next, we conducted an analysis to evaluate the influence of the investigated genotypes on age of dementia diagnosis and duration of disease. Patients with the *ABCA1* (rs2230806) GG variant presented a shorter dementia duration compared to those with the AA and GA genotypes (*p* = 0.0001 and *p* = 0.0004, respectively). Moreover, individuals with the *ABCA1* (rs2230806) GG genotype achieved lower scores on MoCA scale during their first examination (*p* = 0.01) (Figure 1).

An analysis of the impact of the polymorphisms studied on total cholesterol, LDL cholesterol, HDL cholesterol, and triglyceride levels was not conducted due to the long-term use of statins by patients in the hyperlipidemia group.

## 3. Discussion

The aim of this study was to assess the usefulness of *ABCA1*, *ABCB7*, *ABCB1*, *APOE*, *CYP46A1*, and *LRP1* genotyping as promising dementia risk factors among a wide group of patients diagnosed first with hyperlipidemia. Our findings indicate that *ABCA1* (rs2230806) is a suitable candidate. Analysis of other investigated polymorphisms did not reveal differences between the compared groups.

The ATP-binding cassette transporter ABCA1 plays a central role in the maintenance of cellular cholesterol homeostasis by promoting efflux of cholesterol and phospholipids from cells (e.g., hepatocytes, macrophages, and glial cells) to apolipoproteins such as ApoA-I and ApoE, initiating the formation of nascent HDL particles and facilitating reverse cholesterol transport [15,16]. In the periphery, ABCA1 plays a key role in regulating plasma HDL-C, LDL-C, and triglyceride levels, thereby reducing atherogenic lipid accumulation [17].

Genetic polymorphisms in the *ABCA1* gene, including but not limited to rs2230806 (R219K) and rs2230808 (R1587K), have been significantly associated with alterations in plasma lipid profiles. For instance, meta-analyses show that A-allele carriers of rs2230806 exhibit higher HDL-C and lower LDL-C/TG levels [18,19]. In diabetic or dyslipidemic populations, novel variants (e.g., rs2066714 and rs757194699) are linked to increased susceptibility to dyslipidemia through altered ABCA1-apoA1 interaction and impaired lipid efflux capacity [20]. These findings indicate that the *ABCA1* genotype may modulate hyperlipidemia severity by affecting transporter functionality and lipid efflux efficiency [18,20].

In the central nervous system, ABCA1 is essential for lipidation of ApoE, which in turn facilitates clearance of amyloid-β (Aβ) and supports neuronal cholesterol efflux [15,17]. Impaired ABCA1 function (due to polymorphisms or downregulation in disease states) leads to reduced ApoE lipidation, diminished Aβ clearance, and increased Aβ and tau pathology, and accelerates cognitive decline [15,20,21]. Moreover, recent mechanistic studies have shown that intracellular accumulation of cholesterol and oxysterols can mislocalize ABCA1 to lysosomes via caveolin-1, thereby reducing lipid efflux, activating senescence- and mTORC1-associated pathways, and ultimately accelerating neurodegeneration, particularly in *APOE* E4 carriers [16,17].

Within this context, the *ABCA1* genotype functions as a modifier of lipid burden and lipid-driven neurodegeneration: individuals carrying disadvantageous *ABCA1* variants in the presence of dyslipidemia are at increased risk of cholesterol-induced neuronal pathology [18,19,20]. This mechanistic insight underscores the importance of considering both lipid-related genetic factors, such as *ABCA1* polymorphisms, and systemic lipid status, such as hyperlipidemia, when assessing the risk and progression of neurodegenerative diseases and atherosclerotic complications [15,16,17,18,19,20].

Analyses of the rs2230806 polymorphism are most commonly conducted in the background of evaluating coronary artery disease (CAD) susceptibility. Studies have shown that the G allele of rs2230806 is significantly more frequent in coronary artery disease patients, potentially increasing CAD risk [22]. Moreover, a meta-analysis revealed that rs2230806 A allele carriers had higher HDL-C levels and lower LDL-C and triglyceride levels compared to non-carriers [23]. Recent research highlights a strong association between cardiovascular disease and Alzheimer’s disease (AD). Individuals with subclinical CVD are at higher risk for dementia and AD. Common risk factors for both conditions include hypertension, high LDL cholesterol, low HDL cholesterol, and diabetes [24]. In light of this evidence, analysis in relation of neurodegeneration is required. Studies using mice lacking brain *ABCA1* have shown increased neuroinflammation, astrogliosis, and cortical neuronal death [25]. Another survey on mouse models confirmed that *ABCA1* haplodeficency results in increased Aβ levels and cognitive deficits, but the scale is significantly greater in individuals with the ApoE4 variant [26]. It is worth noting that *ABCA1* is transcriptionally regulated by Liver X Receptors (LXR) and Retinoic X Receptors (RXR), presenting potential therapeutic targets for neurodegenerative disorders [27,28,29].

Contrary to the presented results of studies on mice, findings from published genetic association studies on the link between *ABCA1* and Alzheimer’s disease are inconclusive. In a Hungarian report, the minor A allele of rs2230806 was linked to a modest protective effect against Alzheimer’s disease [30]. Similarly, in the Chinese Han population, individuals with the A allele achieved better scores on the MMSE cognitive impairment assessment scale and had significantly higher concentrations of HDL fraction than non-carriers [31]. According to Wavrant-De Vrièze et al., AG and GG genotypes are associated with the onset of Alzheimer’s disease; however, its coincidence with the ApoE variant is noteworthy [32]. Sundar et al. indicate a gender-specific association between the *ABCA1* rs2230806 polymorphism and late-onset Alzheimer’s disease (LOAD) because women with A allele exhibited a 1.75-fold higher risk of the disease [33].

We found a significant difference in *ABCA1* (rs2230806) genotype distribution between the involved groups with hyperlipidemia and dementia vs. with hyperlipidemia. Moreover, GG genotype is related to AD incidence. Patients with the GG genotype in comparison to other variants had shorter dementia duration and lower scores on the MoCA test during the first examination. Until now, many publications have confirmed that the best risk marker for dementia is the *APOE* E4 variant; however, the vast majority have relied on comparative studies of healthy individuals and those with dementia. In our study, both groups of participants included patients with hyperlipidemia as an underlying condition, and the differentiating criterion was the presence or absence of dementia. Considering the essential role of APOE as a primary lipid transporter, this enhances the validity of the conducted experiment.

Currently, neuropathological examination of the brain, neuroimaging, and biomarkers are widely accepted for the diagnosis of dementia, including the measurement of tau protein, phosphorylated tau protein, and Aβ 1–42, which requires the performance of an invasive procedure to collect cerebrospinal fluid, and can provide high diagnostic accuracy in diagnosis of dementia [34]. Some promising plasma phosphorylated tau protein and Aβ42/Aβ40 ratio biomarkers have also been reported recently [35,36].

However, molecular testing lately has become a basic diagnostic tool in primary care and has promising potential to be used in different clinical cases. It is significant to consider the usefulness of genetic markers as screening factors in targeted groups of patients.

### Limitations

The conducted study is characterized by several limitations primarily related to the sample size and the polymorphisms selected based on a review of the global literature, which have been reported as involved in both lipid metabolism disorders and dementia-related diseases. However, this study was intended as a preliminary analysis of the potential for identifying gene polymorphisms that should be considered as markers for dementia development in a dedicated patient group. Increasing the number of cases, conducting the analysis across different populations, and significantly expanding the number of polymorphisms tested, for example, through panel tests, would substantially enhance the reliability of the obtained data. In our study, we also did not take into account laboratory test results, particularly those related to lipid metabolism, due to differences in statin use between the two study groups. Cautious planning and implementation of extended lipid parameter diagnostics would undoubtedly add value by narrowing down the patient groups for which molecular test results could directly influence clinical decisions.

## 4. Materials and Methods

### 4.1. Study Population

The study protocol included 203 patients: 109 diagnosed with both dementia (Alzheimer’s disease, 69, vascular and mixed dementia, 36, frontotemporal dementia, 3, Lewy body dementia, 1) and hyperlipidemia hospitalized in the Department of Psychiatry Wrocław Medical University and 94 with hyperlipidemia from 4 Military Hospital with Polyclinic in Wrocław. Moreover, 101 healthy volunteers were enrolled as a genotype frequency control group. Dementia was diagnosed in line with Alzheimer’s Association criteria while hyperlipidemia was diagnosed according to defined LDL-C levels and risk of cardiovascular disease. Exclusion criteria for the dementia and hyperlipidemia group were (1) brain injuries, (2) acquired causes of dementia, (3) liver or kidney disease, (4) secondary causes of dyslipidemia, and (5) long-term fat-elimination diet. Exclusion criteria for the hyperlipidemia group were (1) dementia, (2) memory changes, (3) cognitive impairments, (4) behavioral and mood changes, (5) decline in daily functioning, (6) liver or kidney disease, (7) secondary causes of dyslipidemia, and 8) long-term fat-elimination diet. Patients were informed about the details of the study and signed fixed consent forms for participation in molecular tests and clinical data processing without personalities. Questionnaires completed by authorized representatives in each department included demographic data (sex and age), anthropometric data (weight), laboratory results (cholesterol, LDL, HDL, and triglycerides), comorbidities (diabetes and hypertension), age of diagnosis, duration of dementia, and neuropsychological assessment including MMSE and MoCA results collected at the early stage of diagnosed cognitive impairment, which subsequently progressed to dementia. The study protocol was approved by the Local Ethics Committee of the Wrocław Medical University (141/2019).

### 4.2. Genotyping

One blood EDTA sample was completed to extract DNA in the commercial kit (Omega Bio-tek, Norcross, GA, USA) for the genetic survey. The participants were genotyped for *ABCA1* (rs2230806, rs2422493), *ABCA7* (rs4147929, rs3752246 rs3764650), *ABCB1* (rs1045642, rs1128503, rs2032582) *APOE*, (rs429358, rs7412), *CYP46A1* (rs754203), and *LRP1* (rs1799986) using AmpliSNiP Dementia Screening Panel (qPCR) (Amplicon, Wrocław, Poland) on Biometra TOptical 96 system (Analytic Jena, Jena, Germany).

### 4.3. Laboratory Markers

Total cholesterol, triglycerides, and HDL cholesterol were measured during standard diagnostic process in hospital using the enzymatic colorimetric method on a biochemical analyzer using commercially available kits (Roche Diagnostics, Mannheim, Germany). LDL concentrations were calculated using the Friedewald formula (in case of triglycerides ≥ 400 mg/dl measured directly).

### 4.4. Statistical Analysis

The statistical analysis was performed using Statistica Ver. 13.3. (TIBCO Software Inc., Palo Alto, CA, USA). The normality of the distribution was evaluated using the Shapiro–Wilk test. The homogeneity of variance was assessed with Levene’s test. Variables with a normal distribution were presented as the mean ± standard deviation, while those with a different distribution were presented as the median along with the first (Q1) and third (Q3) quartile. Qualitative data were expressed as an average percentage. Chi-squared or the Fisher exact test was performed to compare categorical data. ANOVA, Kruskal–Wallis, or Mann–Whitney test was used to compare continuous variables. *p*-value below 0.05 was considered as significant.

## 5. Conclusions

The results of our research confirm the impact of the *ABCA1* gene polymorphism rs2230806 on an increased risk of developing dementia among patients with hyperlipidemia. This polymorphism is also associated with the duration of the disease and MoCA scale results in the early stages of diagnosed mild cognitive impairment, which over time progressed to dementia. The *ABCA1* gene is involved in the regulation of cholesterol transport and phospholipid balance in cells, while the rs2230806 polymorphism leads to downregulation of gene activity, playing a significant role in the development of atherosclerosis and other age-related diseases. This may suggest that changes also occur within the central nervous system, promoting the onset of dementia. However, due to the relatively small sample size in this experiment, it is necessary to expand the study group in order to confirm the utility of *ABCA1* rs2230806 genotyping as a predictive marker for the onset of dementia in individuals with hyperlipidemia.

## Figures and Tables

**Table 1:** Genes under investigation and their role in lipid metabolism and dementia.

Gene	Role in Lipid Metabolism	Involvement in Dementia	References
*ABCA1*	Regulation of cholesterol, support apolipoprotein E (ApoE) lipidation	Influence on ApoE lipidation affecting amyloid precursor protein (APP) processing and beta-amyloid (Aβ) cumulation	[6,7]
*ABCA7*	Regulation of lipid metabolism, apolipoprotein lipidation	Phagocytic elimination of Aβ, suppression of APP proteolysis	[6,7,8]
*ABCB1*	Efflux transporter, pharmacoresistance, detoxification, homeostasis maintenance in central nervous system (CNS)	Transport of Aβ from neural processes to blood circulation	[6]
*APOE*	Lipid transporter protein	*APOE* E4 variant accelerates the accumulation, aggregation, and deposition of Aβ in the brain	[9]
*CYP46A1*	Conversion cholesterol into form 24S-hydroxycholesterol (24S-OHC) capable of crossing blood–brain barrier	Decreased function leads to an increased production of Aβ and tau proteins	[10,11,12]
*LRP1*	Internalization of lipoproteins into neurons, ApoE receptor	Aβ deposition, tau regulation, brain homeostasis	[13,14]

**Table 2:** Attributes of the sample.

Parameter	Dementia and Hyperlipidemia (*n* = 109)	Hyperlipidemia (*n* = 94)	*p*-Value
Male/Female, sex	47/62 (43.1%/56.9%)	37/57 (39.4%/60.6%)	*p* = 0.588
Age, y	70.8 (±11.5)	64.1 (±12.9)	*p* < 0.05
Weight, kg	78.0 (70.0 ÷ 86.5)	79.5 (68.0÷95.5)	*p* = 0.420
Diabetes	13 (11.9%)	27 (28.7%)	*p* < 0.05
Hypertension	20 (18.4%)	68 (72.3%)	*p* < 0.05
Age of dementia diagnosis, y	65.8 (±9.8)	-	
Dementia duration, y	7.8 (±2.4)	-	
MMSE score	18.1 (±4.0)	-	
MoCA score	19.0 (±4.1)	-	
Total cholesterol, mg/dL	231.0 (220.0 ÷ 244.0)	146.0 (118.0 ÷ 168.0)	*p* < 0.05
HDL cholesterol, mg/dL	55.0 (49.0 ÷ 64.0)	43.0 (32.0 ÷ 56.0)	*p* < 0.05
LDL cholesterol, mg/dL	140.7 (±21.3)	77.3 (±34.6)	*p* < 0.05
Triglicerydes, mg/dL	195.2 (±53.1)	130.7 (±75.5)	*p* < 0.05

Data expressed as *n* (%) mean (SD) or median (first ÷ third quartile). SI conversion: cholesterol to mmol/L, multiplied by 0.0259; triglycerides to mmol/L, by 0.0113.

**Table 3:** Genotype frequency.

Gene	rs Code	Participants	Genotype Frequency						*p*-Value
*ABCA1*	rs2230806		AA		GA		GG		0.004
		Dementia and hyperlipidemia	39 (35.8%)		36 (33.0%)		34 (31.2%)		
		Hyperlipidemia	45 (47.8%)		40 (42.6%)		9 (9.6%)		
		Controls	37 (36.6%)		44 (43.6%)		20 (19.8%)		
*ABCA1*	rs2422493		CC		CT		TT		0.066
		Dementia and hyperlipidemia	34 (31.2%)		51 (46.8%)		24 (22.0%)		
		Hyperlipidemia	20 (21.3%)		56 (59.6%)		18 (19.1%)		
		Controls	20 (19.8%)		49 (48.5%)		32 (31.7%)		
*ABCA7*	rs4147929		AA		AG		GG		0.561
		Dementia and hyperlipidemia	3 (2.8%)		35 (32.1%)		71 (65.1%)		
		Hyperlipidemia	2 (2.1%)		30 (31.9%)		62 (66.0%)		
		Controls	3 (3.0%)		23 (22.8%)		75 (74.2%)		
*ABCA7*	rs3752246		CC		CG		GG		0.493
		Dementia and hyperlipidemia	71 (65.1%)		36 (33.0%)		2 (1.9%)		
		Hyperlipidemia	64 (68.1%)		29 (30.8%)		1 (1.1%)		
		Controls	77 (76.2%)		23 (22.8%)		1 (1.0%)		
*ABCA7*	rs3764650		GG		GT		TT		0.446
		Dementia and hyperlipidemia	1 (0.9%)		17 (15.6%)		91 (83.5%)		
		Hyperlipidemia	1 (1.1%)		11 (11.7%)		82 (87.2%)		
		Controls	-		10 (9.9%)		91 (90.1%)		
*ABCB1*	rs1045642		CC		CT		TT		0.478
		Dementia and hyperlipidemia	27 (24.8%)		55 (50.4%)		27 (24.8%)		
		Hyperlipidemia	28 (29.8%)		44 (46.8%)		22 (23.4%)		
		Controls	19 (18.8%)		52 (51.5%)		30 (29.7%)		
*ABCB1*	rs1128503		CC		CT		TT		0.512
		Dementia and hyperlipidemia	38 (34.9%)		48 (44.0%)		23 (21.1%)		
		Hyperlipidemia	38 (40.4%)		48 (43.6%)		15 (16.0%)		
		Controls	30 (29.7%)		47 (46.5%)		24 (23.8%)		
*ABCB1*	rs2032582		GG		GT		TT		0.353
		Dementia and hyperlipidemia	36 (33.6%)		51 (47.7%)		20 (18.7%)		
		Hyperlipidemia	44 (46.8%)		37 (39.4%)		13 (13.8%)		
		Controls	34 (33.7%)		49 (48.5%)		18 (17.8%)		
*APOE*	rs429358 rs7412		E2/E2	E2/E3	E2/E4	E3/E3	E3/E4	E4/E4	0.224
		Dementia and hyperlipidemia	-	16 (14.7%)	5 (4.6%)	77 (70.6%)	7 (6.4%)	4 (3.7%)	
		Hyperlipidemia	1 (1.1%)	6 (6.3%)	1 (1.1%)	67 (71.3%)	19 (20.2%)	-	
		Controls	1 (1.0%)	7 (6.9%)	1 (1.0%)	86 (85.2%)	6 (5.9%)	-	
*CYP46A1*	rs754203		AA		AG		GG		0.148
		Dementia and hyperlipidemia	51 (46.8%)		52 (47.7%)		6 (5.5%)		
		Hyperlipidemia	44 (46.8%)		35 (37.2%)		15 (16.0%)		
		Controls	48 (47.5%)		40 (39.6%)		13 (12.9%)		
*LRP1*	rs1799986		CC		CT		TT		0.224
		Dementia and hyperlipidemia	75 (68.8%)		31 (28.4%)		3 (2.8%)		
		Hyperlipidemia	73 (77.6%)		17 (18.1%)		4 (4.3%)		
		Controls	65 (64.4%)		32 (31.6%)		4 (4.0%)		

## Data Availability

The data presented in this study are available on request from the corresponding author due to (The limitation in data access arises from the nature of the work, which involves the comparison of genetic test results. The results of genetic tests are protected under medical confidentiality, the General Data Protection Regulation and Polish law. Patients have provided consent for genetic testing by signing an informed consent declaration; however, the test results may only be used for scientific purposes and cannot be disclosed to third parties. The only additional data that may be disclosed to interested parties pertain to the genotyping method or the conduct of statistical analysis).

## Abbreviations

The following abbreviations are used in this manuscript:

24S-OHC	24(S)-Hydroxycholesterol
AD	Alzheimer’s disease
ApoE	Apolipoprotein E
APP	Amyloid precursor protein
Aβ	Amyloid β
BMI	Body mass index
CAD	Coronary artery disease
CNS	Central nervous system
CVD	Cardiovascular disease
HDL	High density lipoprotein
LDL	Low density lipoprotein
LOAD	Late-onset Alzheimer’s disease
MMSE	Mini–Mental State Examination
MoCA	Montreal Cognitive Assessment
WHO	World Health Organization
